# TEAM: A time‐enhanced attention‐based model for virus mutation prediction

**DOI:** 10.1002/qub2.70049

**Published:** 2026-08-16

**Authors:** Jie Ji, Jie Hu, Tianwei Yu, Xiaodan Fan

**Affiliations:** ^1^ Department of Statistics and Data Science The Chinese University of Hong Kong Hong Kong China; ^2^ School of Mathematical Science Xiamen University Xiamen China; ^3^ School of Data Science The Chinese University of Hong Kong‐Shenzhen Shenzhen China

**Keywords:** attention‐based model, deep learning, mutation prediction, phylogenetics, protein language model

## Abstract

The evolution of severe acute respiratory syndrome coronavirus 2 (SARS‐CoV‐2) during the coronavirus disease 2019 (COVID‐19) pandemic highlights the critical need for predicting virus mutations in order to stay ahead of infectious diseases. Here, we present the time‐enhanced attention‐based model (TEAM), which combines phylogenetic sampling with deep learning to improve virus mutation prediction accuracy. TEAM introduces a novel time‐enhanced phylogenetic sampling strategy that preserves both evolutionary and temporal sequence relationships, enhancing its ability to predict site‐specific mutations as a multi‐class classification task. The framework leverages evolutionary scale modeling embeddings and a dual‐attention mechanism to capture sequence‐level patterns and temporal dynamics. Experiments on the SARS‐CoV‐2 spike protein dataset show that TEAM substantially outperforms existing methods. Further evaluations on membrane protein and H1N1 datasets confirm its robustness and generalizability. In practice, TEAM provides a scalable and interpretable solution for mutation prediction, offering valuable insights for evolutionary research and public health planning.

## 1 INTRODUCTION

The coronavirus disease 2019 (COVID‐19) pandemic, caused by severe acute respiratory syndrome coronavirus 2 (SARS‐CoV‐2), introduced unprecedented challenges to global public health, economic stability, and societal well‐being. While global vaccination efforts were effective in reducing infection rates, the continuous emergence of new SARS‐CoV‐2 variants diminished the long‐term efficacy of existing vaccines and therapeutics. Variants driven by mutations, particularly in the spike protein, exhibited changes in transmissibility, immune escape, and increased virulence in some cases. Accurate mutation prediction is crucial for tracking viral evolution, anticipating future outbreaks, and designing effective vaccines and therapeutics.

Since the beginning of the COVID‐19 pandemic, numerous studies have explored various aspects of SARS‐CoV‐2, from diagnostic methods using chest X‐rays and computed tomography scans [1, 2, 3] to machine learning models predicting case numbers and mortality rates [4, 5, 6]. While much of this research have focused on disease diagnosis, transmission modeling, and intervention strategies, only a limited number of studies have investigated mutation prediction at specific protein sites [7, 8]. Existing mutation prediction frameworks largely rely on deep learning models designed for time series data, such as recurrent neural networks (RNN) [9], long short‐term memory (LSTM) [10], and Transformer [11]. These models have significantly advanced our ability to model sequential data and predict mutation trends. In parallel, the rapid development of protein language models (PLMs), such as ProtTrans [12], evolutionary scale modeling (ESM) [13], and AlphaFold embeddings [14], has opened new avenues for extracting rich contextual representations from protein sequences. PLMs, pre‐trained on massive protein datasets, capture both local and global dependencies, providing a detailed representation of amino acid interactions that can complement traditional time‐series models.

However, the design and quality of the existing prediction framework, including sampling strategies and feature representations, play a more critical role in achieving robust and accurate predictions. For instance, the TEMPEL framework [7] uses clustering techniques to group amino acid sequences by similarity across years, representing evolutionary nodes with branches. While effective for influenza, which evolves gradually over long periods, this approach is less suitable for short‐term, rapidly evolving viruses such as SARS‐CoV‐2. Conversely, the TEMPO framework [8] employs phylogenetic tree (PT)‐based sampling to extract historical sequences along evolutionary paths, offering a more realistic view of mutation dynamics. However, TEMPO struggles with short evolutionary paths and lacks temporal information, which is critical for capturing seasonal or annual trends. Moreover, most frameworks are designed for binary classification tasks (predicting whether a mutation occurs) rather than multi‐class classification tasks (identifying specific amino acid substitutions), limiting their utility in fine‐grained mutation prediction. Furthermore, the incorporation of PLMs in existing frameworks remains limited, despite their demonstrated effectiveness in tasks such as protein structure prediction and function annotation, and their potential to model sequence dependencies and capture evolutionary contexts for mutation prediction, which has yet to be fully utilized.

To address these limitations, we propose a novel time‐enhanced attention‐based model (TEAM), a mutation prediction framework that integrates advanced sampling, feature representation, and predictive modeling. The framework comprises three key components. First, the time‐enhanced phylogenetic sampling method preserves complete evolutionary paths while imposing temporal constraints to efficiently capture amino acid mutations over specific intervals. By combining PT‐based sampling with temporal information, this sampling method is particularly effective for short‐term, high‐mutation‐rate viruses such as SARS‐CoV‐2. Second, we employ embeddings from the popular PLM ESM [13], which capture both local and global sequence dependencies and provide richer feature representations than traditional ProtVec embeddings [15], thereby enhancing mutation prediction. Third, we adopt an enhanced dual‐attention prediction model based on dual‐attention recurrent neural network (DA‐RNN) [16], incorporating input and temporal attention mechanisms. The model is modified for multi‐class classification, enabling prediction of specific amino acid substitutions, and integrates time embeddings to capture temporal trends (e.g., quarterly or yearly mutation patterns). Extensive experiments on SARS‐CoV‐2 spike protein datasets demonstrate that our framework outperforms state‐of‐the‐art methods in predicting mutation dynamics. Its robustness is further validated on the SARS‐CoV‐2 membrane protein and the hemagglutinin (HA) and neuraminidase (NA) proteins of the 2009 pandemic influenza virus (H1N1pdm09).

Main results of real data analyses and further evaluations are shown in Section 2. Discussions are presented in Section 3. The SARS‐CoV‐2 spike protein datasets and the entire framework are elaborated in Section 4.

## 2 RESULTS

### 2.1 Experiment setup

#### 2.1.1 Data preparation

After data cleaning, the resulting dataset comprised 53,726 full‐length spike protein sequences, each 1273 amino acids long, segmented by quarterly intervals spanning from 2020Q1 to 2022Q2. A total of 672 unique evolutionary paths were identified. From each path, 50 samples were drawn, and duplicates were removed, resulting in 13,407 time‐ordered sequence samples. Subsequently, mutated sites within each sequence and an equal number of nonmutated sites were identified, leading to a final set of 7196 site‐specific samples after deduplication. This dataset was split into training and testing sets in a 4:1 ratio and ensure all time points in the training set precede those in the testing set to simulate real‐world prediction scenarios. The prediction task involves multi‐class amino acid substitution, and the dataset was balanced to maintain an equal number of mutated and non‐mutated cases at each site, ensuring robust performance evaluation. To account for variability in the number of samples across evolutionary paths, the dataset was standardized by selecting the last *k* = 5 nonempty time points from each path, ensuring consistency with other methods.

#### 2.1.2 Baseline models

For comparison, we evaluated our framework against multiple baselines across three dimensions:

- **Sampling methods**: We compared our time‐enhanced phylogenetic sampling with two alternative sampling methods: cluster + time and PT‐based sampling.
- **Embedding techniques**: We contrasted the performance of ESM‐1v embeddings with 3‐g + ProtVec embeddings to evaluate the impact of feature representation.
- **Prediction models**: The baseline models included RNN, LSTM, gated recurrent unit (GRU) [17], Attention [18], Transformer, DA‐RNN, and our proposed T‐DARNN. These models were benchmarked to assess their performance in amino acid mutation prediction tasks.

#### 2.1.3 Evaluation metrics

To evaluate the performance of our model, we use standard metrics for multi‐class classification, including accuracy, precision, recall, F1‐score, and Matthews correlation coefficient (MCC). These metrics are calculated across all classes using three different averaging methods: macro‐averaging, micro‐averaging, and weighted‐averaging. Macro‐averaging calculates the metric independently for each class and then takes an unweighted average across all classes, while micro‐averaging aggregates the contributions of all classes to compute the metrics, and weighted‐averaging accounts for the support of each class.

Since our task ensures a balance in mutation versus nonmutation cases, we prioritize micro‐averaged metrics for their robustness to class imbalance. Additionally, because micro‐averaged precision, recall, and F1‐score are identical in this case, we report only accuracy and MCC to avoid redundancy while emphasizing metrics most relevant to mutation prediction tasks. The overall accuracy is calculated as

(1) $$\begin{array}{c} \text{Accuracy}=\frac{\sum_{k=1}^{K}{\text{TP}}_{k}}{\text{Total}\;\text{samples}} \end{array},$$

here ${\text{TP}}_{k}$ represents the number of true positives regarding to k‐th class. The micro‐averaged MCC in multi‐class tasks is defined as

(2) $$\begin{array}{c} \text{MCC}=\frac{\sum_{i=1}^{K}\sum_{j=1}^{K}\left(C_{ii}\cdot C_{jj}-C_{ij}\cdot C_{ji}\right)}{\sqrt{\left(\sum_{i=1}^{K}R_{i}\right)\left(\sum_{i=1}^{K}C_{i}\right)\left(\sum_{i=1}^{K}R_{i}-C_{ii}\right)\left(\sum_{i=1}^{K}C_{i}-C_{ii}\right)}}, \end{array}$$

where $C_{ij}$ is the confusion matrix entry for true class i predicted as j, $R_{i}$ is the sum of the i‐th row, and $C_{i}$ is the sum of the i‐th column.

### 2.2 Effectiveness

In this section, we evaluate the effectiveness of our proposed framework for predicting amino acid mutations in the SARS‐CoV‐2 spike protein. The experiments compare different sampling methods, embedding techniques, and deep learning models to highlight the advantages of our framework. The results are summarized in Tables 1 and 2.

**TABLE 1:** Comparison of sampling methods using 3‐g + ProtVec embedding on SARS‐CoV‐2 S protein.

Sampling method	Model	ACC	MCC
Cluster + time	RNN	0.507 ± 0.005	0.477 ± 0.005
	LSTM	0.517 ± 0.004	0.488 ± 0.004
	GRU	0.508 ± 0.004	0.480 ± 0.004
	Attention	0.514 ± 0.003	0.485 ± 0.003
	DA‐RNN	0.503 ± 0.004	0.473 ± 0.004
	Transformer	0.509 ± 0.003	0.481 ± 0.03
PT‐based	RNN	0.633 ± 0.016	0.611 ± 0.017
	LSTM	0.666 ± 0.012	0.645 ± 0.012
	GRU	0.652 ± 0.009	0.631 ± 0.009
	Attention	0.671 ± 0.007	0.650 ± 0.007
	DA‐RNN	0.617 ± 0.024	0.592 ± 0.024
	Transformer (TEMPO)	0.649 ± 0.010	0.620 ± 0.010
**Time‐enhanced PT**	RNN	0.772 ± 0.005	0.756 ± 0.005
	LSTM	0.782 ± 0.008	0.767 ± 0.008
	GRU	0.784 ± 0.005	0.768 ± 0.005
	Attention	0.782 ± 0.009	0.763 ± 0.009
	DA‐RNN	0.772 ± 0.007	0.753 ± 0.007
	Transformer	0.779 ± 0.008	1.758 ± 0.008

*Note*: Bold entries denote the optimal performance with the highest ACC and MCC values within each model group. These abbreviations and formatting rules apply consistently to all subsequent tables throughout the manuscript.

Abbreviations: ACC, accuracy; MCC, Matthews correlation coefficient; PT, phylogenetic tree.

**TABLE 2:** Comparison of embedding methods and models on SARS‐CoV‐2 S protein.

Embedding	Model	ACC	MCC
3‐G + ProtVec	RNN	0.772 ± 0.005	0.756 ± 0.005
	LSTM	0.782 ± 0.008	0.767 ± 0.008
	GRU	0.784 ± 0.005	0.768 ± 0.005
	Attention	0.782 ± 0.009	0.763 ± 0.009
	DA‐RNN	0.772 ± 0.007	0.753 ± 0.007
	Transformer	0.779 ± 0.008	0.758 ± 0.008
	T‐DARNN	0.773 ± 0.006	0.754 ± 0.006
**ESM‐1v**	RNN	0.835 ± 0.007	0.823 ± 0.007
	LSTM	0.837 ± 0.007	0.825 ± 0.008
	GRU	0.838 ± 0.007	0.826 ± 0.007
	Attention	0.837 ± 0.006	0.825 ± 0.007
	DA‐RNN	0.846 ± 0.002	0.831 ± 0.008
	Transformer	0.839 ± 0.008	0.827 ± 0.009
	**T‐DARNN (TEAM)**	**0.849 ± 0.005**	**1.835 ± 0.002**

*Note*: Bold entries denote the optimal performance with the highest ACC and MCC values within each model group.

Abbreviations: ACC, accuracy; MCC, Matthews correlation coefficient; T‐DARNN, (time‐enhanced DA‐RNN) denotes the model proposed in TEAM.

Table 1 compares three sampling methods: time series clustering (TEMPEL), PT‐based (TEMPO), and time‐enhanced PT (TEAM), using 3‐g + ProtVec embeddings across five baseline models. As shown in the table, the PT‐based method improves upon the clustering approach, emphasizing the importance of incorporating phylogenetic relationships on spike protein dataset. For instance, the accuracy of the DA‐RNN model increases from 0.503 to 0.617, and the MCC improves from 0.473 to 0.592.

Our proposed TEAM method further enhances performance by integrating temporal information during sampling. Compared to TEMPEL, TEAM achieves substantial gains across all models. For example, the DA‐RNN model’s accuracy improves by 26.9% (from 0.503 to 0.772), and its MCC improves by 28.0% (from 0.473 to 0.753). These results demonstrate that TEAM effectively captures the evolutionary and temporal dynamics of the SARS‐CoV‐2 S protein, enabling more accurate mutation predictions.

Table 2 examines the impact of embedding methods (3‐g + ProtVec vs. ESM‐1v) and models on prediction performance, using our time‐enhanced PT‐based sampling method. ESM‐1v embeddings consistently outperform 3‐g + ProtVec, owing to their ability to capture rich structural and evolutionary information. In this overall improvement from TEMPO to TEAM, the introduction of ESM‐1v embedding alone contributes around 30% of the total accuracy gain and total MCC gain, demonstrating its critical role in performance enhancement.

Additionally, the enhanced DA‐RNN model in our TEAM framework, which incorporates time embeddings with ESM‐1v, achieves the best overall performance. It reaches an accuracy of 0.849 and an MCC of 0.835, outperforming all baseline models. This result highlights the effectiveness of combining advanced embedding methods with a temporal‐aware model design to address the challenges of mutation prediction in SARS‐CoV‐2.

Key observations are as follows:

- Incorporating temporal information during sampling (TEAM) significantly improves model performance, as it captures the evolutionary history and temporal dynamics of the SARS‐CoV‐2 S protein.
- ESM‐1v embeddings provide a substantial advantage over traditional 3‐g + ProtVec embeddings, demonstrating the value of pre‐trained PLMs.
- The enhanced DA‐RNN model in TEAM achieves the best results across all metrics, confirming the effectiveness of integrating time‐aware embeddings with advanced attention mechanisms.

### 2.3 Robustness

#### 2.3.1 SARS‐CoV‐2 membrane protein

To further evaluate the robustness and generalizability of our proposed framework across SARS‐CoV‐2 proteins, we conducted experiments on the membrane protein using the same evaluation setup as the spike protein. The results include comparisons of sampling methods (Table 3) and embedding methods with various models (Table 4).

**TABLE 3:** Comparison of sampling methods using 3‐g + ProtVec embedding on SARS‐CoV‐2 M protein.

Sampling method	Model	ACC	MCC
Cluster + time	RNN	0.646 ± 0.004	0.620 ± 0.003
	LSTM	0.641 ± 0.001	0.614 ± 0.001
	GRU	0.639 ± 0.004	0.612 ± 0.005
	Attention	0.639 ± 0.004	0.613 ± 0.004
	DA‐RNN	0.640 ± 0.004	0.613 ± 0.004
	Transformer	0.637 ± 0.003	0.612 ± 0.005
PT‐based	RNN	0.834 ± 0.006	0.828 ± 0.006
	LSTM	0.843 ± 0.006	0.837 ± 0.006
	GRU	0.837 ± 0.016	0.828 ± 0.016
	Attention	0.846 ± 0.005	0.840 ± 0.005
	DA‐RNN	0.840 ± 0.007	0.832 ± 0.007
	Transformer (TEMPO)	0.841 ± 0.007	0.832 ± 0.007
**Time‐enhanced PT**	RNN	0.901 ± 0.005	0.896 ± 0.005
	LSTM	0.906 ± 0.003	0.902 ± 0.003
	GRU	0.905 ± 0.002	0.902 ± 0.002
	Attention	0.906 ± 0.003	0.902 ± 0.003
	DA‐RNN	0.887 ± 0.009	0.880 ± 0.009
	Transformer	0.897 ± 0.006	0.883 ± 0.006

*Note*: Bold entries denote the optimal performance with the highest ACC and MCC values within each model group.

Abbreviations: ACC, accuracy; MCC, Matthews correlation coefficient; PT, phylogenetic tree.

**TABLE 4:** Comparison of embedding methods and models on SARS‐CoV‐2 M protein.

Embedding	Model	ACC	MCC
3‐G + ProtVec	RNN	0.901 ± 0.005	0.896 ± 0.005
	LSTM	0.906 ± 0.003	0.902 ± 0.003
	GRU	0.905 ± 0.002	0.902 ± 0.002
	Attention	0.906 ± 0.003	0.902 ± 0.003
	DA‐RNN	0.887 ± 0.009	0.880 ± 0.009
	Transformer	0.897 ± 0.006	0.883 ± 0.006
	T‐DARNN	0.889 ± 0.009	0.881 ± 0.009
**ESM‐1v**	RNN	0.925 ± 0.004	0.920 ± 0.004
	LSTM	0.927 ± 0.004	0.923 ± 0.004
	GRU	0.918 ± 0.006	0.913 ± 0.006
	Attention	0.916 ± 0.013	0.910 ± 0.014
	DA‐RNN	0.924 ± 0.003	0.919 ± 0.003
	Transformer	0.921 ± 0.004	0.915 ± 0.004
	**T‐DARNN (TEAM)**	**0.934 ± 0.007**	**0.929 ± 0.007**

*Note*: Bold entries denote the optimal performance with the highest ACC and MCC values within each model group.

Abbreviations: ACC, accuracy; MCC, Matthews correlation coefficient; T‐DARNN, (time‐enhanced DA‐RNN) denotes the model proposed in TEAM.

Table 3 demonstrates that the time‐enhanced PT‐based sampling method consistently outperforms the cluster + time (TEMPEL) and PT‐based (TEMPO) methods across all models, consistent with the trends observed for the S protein. The time‐enhanced PT method achieves the highest accuracy and MCC values across models, with significant performance improvements when combined with advanced models like LSTM and GRU. These results confirm the robustness of the proposed sampling strategy in capturing relevant evolutionary signals for mutation prediction.

In Table 4, models using ESM‐1v embeddings outperform those using 3‐g + ProtVec embeddings, as expected. Similarly, compared with the TEMPO baseline, the ESM‐1v embedding component contributes approximately 25% of the total accuracy improvement and 33.0% of the total MCC improvement in our TEAM framework.

Among the tested models, the enhanced DA‐RNN model achieves the best performance, which demonstrates that when our complete pipeline (TEAM) is applied, it effectively integrates advanced sampling, embedding, and model designs to produce the best results.

When comparing the results on the M protein to those on the S protein, we observe consistent trends in the effectiveness of the proposed framework. The time‐enhanced PT sampling method and ESM‐1v embeddings provide significant improvements in accuracy and MCC across both proteins, demonstrating the framework’s robustness and adaptability to different SARS‐CoV‐2 proteins. This consistency underscores the ability of our method to generalize well to different protein types within the SARS‐CoV‐2 virus, making it a versatile solution for mutation prediction tasks.

#### 2.3.2 Pandemic H1N1/09 HA and NA proteins

To evaluate the robustness of our framework beyond SARS‐CoV‐2, we conducted experiments on the hemagglutinin (HA) and neuraminidase (NA) proteins of the influenza A (H1N1) pdm09 virus. Unlike SARS‐CoV‐2, which emerged as a rapidly mutating virus with numerous branches during its relatively short‐lived outbreaks, influenza H1N1pdm09 represents a long‐standing virus with fewer branches and more stable evolutionary dynamics. These fundamental differences pose unique challenges for sampling strategies and model evaluation, making H1N1pdm09 an ideal candidate for assessing the adaptability of our framework.

Due to the short evolutionary paths in the PT of H1N1pdm09, it was not feasible to apply PT‐based sampling methods. Instead, clustering‐based sampling was used to construct the dataset. However, as shown in Table 5, the clustering‐based sampling approach inherently suffers from significant class imbalance, where positive samples are heavily under‐represented. This imbalance affects the performance of binary classification models, resulting in low recall, F1‐score, and MCC across all tested models. The inherent imbalance demonstrates the limitations of clustering‐based sampling when applied to datasets with uneven class distributions.

**TABLE 5:** Binary classification results using clustering‐based sampling on H1N1pdm09 proteins.

Metric	RNN	LSTM	GRU	Attention	DA‐RNN	Transformer
Accuracy	0.965	0.968	0.967	0.963	0.963	0.963
Precision	0.667	0.786	0.818	0.714	0.714	0.714
Recall	0.250	0.275	0.225	0.125	0.125	0.125
F1‐score	0.364	0.407	0.353	0.213	0.213	0.213
MCC	0.395	0.453	0.419	0.289	0.289	0.289

Moreover, applying clustering‐based sampling to multi‐class mutation prediction showed inflated evaluation metrics because of the imbalanced distribution of amino acid categories. Models tended to favor predictions of no mutations for most sites due to class imbalance, resulting in artificially high accuracy that did not reflect realistic biological outcomes. In contrast, the robustness of our TEAM framework lies in its ability to handle imbalanced and complex datasets, making it a more versatile solution for mutation prediction.

Tables 6 and 7 present the performance of different embedding methods and models for HA and NA proteins, respectively. Models using ESM‐1v embeddings consistently outperformed those using 3‐g + ProtVec embeddings. While the DA‐RNN model generally performed well, it did not achieve the top performance across HA and NA datasets.

**TABLE 6:** Comparison of models on H1N1pdm09 HA protein.

Embedding	Model	ACC	MCC
3‐G + ProtVec	RNN	0.575 ± 0.009	0.543 ± 0.009
	LSTM	0.570 ± 0.005	0.537 ± 0.005
	GRU	0.578 ± 0.006	0.543 ± 0.005
	Attention	0.568 ± 0.013	0.537 ± 0.013
	DA‐RNN	0.561 ± 0.006	0.528 ± 0.006
	Transformer	0.564 ± 0.014	0.539 ± 0.011
	T‐DARNN	0.559 ± 0.007	0.525 ± 0.007
**ESM‐1v**	**RNN**	**0.612 ± 0.006**	**0.584 ± 0.006**
	LSTM	0.603 ± 0.005	0.575 ± 0.005
	GRU	0.609 ± 0.007	0.584 ± 0.007
	Attention	0.606 ± 0.010	0.582 ± 0.010
	DA‐RNN	0.605 ± 0.006	0.571 ± 0.006
	Transformer	0.604 ± 0.005	0.577 ± 0.006
	T‐DARNN (TEAM)	0.609 ± 0.008	0.576 ± 0.009

*Note*: Bold entries denote the optimal performance with the highest ACC and MCC values within each model group.

Abbreviations: ACC, accuracy; MCC, Matthews correlation coefficient; T‐DARNN, (time‐enhanced DA‐RNN) denotes the model proposed in TEAM.

**TABLE 7:** Comparison of models on H1N1pdm09 NA protein.

Embedding	Model	ACC	MCC
3‐G + ProtVec	RNN	0.674 ± 0.003	0.643 ± 0.003
	LSTM	0.678 ± 0.004	0.647 ± 0.004
	GRU	0.681 ± 0.003	0.651 ± 0.003
	Attention	0.682 ± 0.002	0.652 ± 0.002
	DA‐RNN	0.673 ± 0.005	0.641 ± 0.005
	Transformer	0.679 ± 0.003	0.649 ± 0.002
	T‐DARNN	0.671 ± 0.005	0.639 ± 0.005
**ESM‐1v**	RNN	0.714 ± 0.005	0.687 ± 0.006
	**LSTM**	**0.723 ± 0.003**	**0.696 ± 0.004**
	GRU	0.718 ± 0.003	0.691 ± 0.003
	Attention	0.720 ± 0.003	0.690 ± 0.003
	DA‐RNN	0.716 ± 0.002	0.688 ± 0.002
	Transformer	0.719 ± 0.003	0.689 ± 0.002
	T‐DARNN (TEAM)	0.720 ± 0.003	0.690 ± 0.003

*Note*: Bold entries denote the optimal performance with the highest ACC and MCC values within each model group.

Abbreviations: ACC, accuracy; MCC, Matthews correlation coefficient.

For the H1N1pdm09 HA and NA proteins (Tables 6 and 7), the results demonstrate the overall effectiveness of the TEAM framework. For the HA dataset, the RNN achieved the highest accuracy and MCC, reaching 0.612 ± 0.006 and 0.584 ± 0.006, respectively. For the NA dataset, the LSTM model achieved the best performance, with an accuracy and MCC of 0.723 ± 0.003 and 0.696 ± 0.004, respectively. These findings emphasize the strong performance of simpler models in certain scenarios, given the specific characteristics of the H1N1pdm09 proteins.

The enhanced DA‐RNN model within the TEAM framework produced competitive results but did not surpass the simpler models for these datasets. This outcome can be attributed to the unique characteristics of H1N1pdm09 proteins, including limited sequence diversity and inherent sampling challenges, which may reduce the benefits of more complex models like dual attention. Such results are not unexpected, as the effectiveness of complex models often depends on the richness and variability of the underlying data.

Nevertheless, the TEAM framework as a whole, leveraging the ESM‐1v embeddings and an optimized sampling strategy, showcased robust performance across both datasets. The results further validate the versatility of the framework in adapting to distinct evolutionary and structural characteristics of viral proteins, even in challenging cases like H1N1pdm09.

## 3 DISCUSSION AND CONCLUSION

### 3.1 Importance of dataset construction in mutation prediction

The construction of datasets plays a pivotal role in mutation prediction studies. In our evaluation, we observed that issues like temporal data leakage and class imbalance could significantly distort model performance metrics. For instance, in prior approaches such as TEMPEL and TEMPO, temporal data leakage, where test samples were not strictly temporally separated from training samples, led to artificially high accuracy scores, sometimes exceeding 0.99. Additionally, severe class imbalance, as seen in clustering‐based sampling, resulted in inflated precision and accuracy at the cost of recall and MCC. This highlights the critical need for carefully designed sampling methods that align with realistic evolutionary and temporal dynamics.

Our proposed time‐enhanced PT‐based sampling addresses these challenges by enforcing a strict temporal separation between training and testing sets, introducing binary class balance constraints within multi‐classification tasks, and incorporating phylogenetic relationships. This approach enhances the reliability of performance evaluations and establishes a more rigorous standard for dataset construction in future mutation prediction studies.

### 3.2 Limitations of the framework

Despite its promising results, our framework faces certain limitations that warrant discussion:

#### 3.2.1 Dataset dependency

The performance in mutation prediction tasks, including our framework, was significantly influenced by various dataset characteristics, such as raw sequence quality, dataset size, temporal sequence length, and mutation frequency. While our approach ensured consistency within datasets for the same virus, results across different viruses, such as SARS‐CoV‐2 and influenza A‐H1N1pdm09, exhibited notable variability. This variability underscores a broader challenge in mutation prediction studies, highlighting the importance of developing more robust regularization techniques or hybrid architectures to improve generalization across diverse datasets. Future work could explore these directions to enhance the adaptability and reliability of mutation prediction methodologies.

#### 3.2.2 Optimization challenges

As with many deep learning‐based approaches, the process of hyperparameter tuning in our framework proved to be both intricate and resource‐intensive. Despite utilizing Bayesian optimization across 10 runs and averaging the results to ensure reliability, some variability in outcomes persisted, which could impact the reproducibility of results. This highlights a broader challenge in mutation prediction studies, where hyperparameter optimization can significantly influence performance but often requires substantial computational resources. Future work could focus on developing more efficient and automated hyperparameter tuning techniques, or leveraging transfer learning to reduce the need for extensive optimization, thereby improving reproducibility and accessibility in resource‐constrained environments.

### 3.3 Conclusion

In this study, we introduced the TEAM, a comprehensive and innovative framework designed for viral mutation prediction. TEAM seamlessly integrates three core advancements: a time‐enhanced PT‐based sampling method that leverages both temporal and phylogenetic information, advanced embeddings such as ESM‐1v for capturing intricate sequence‐level features, and an enhanced DA‐RNN model with time embedding to incorporate temporal dynamics effectively. By addressing the challenges posed by diverse viral datasets, TEAM excels at capturing complex evolutionary patterns in rapidly mutating viruses like SARS‐CoV‐2 as well as long‐term stable viruses such as influenza A. The framework’s demonstrated adaptability and consistent performance across various viral contexts underscore its potential as a powerful tool for understanding viral evolution, enabling more accurate mutation prediction, and contributing to global efforts in viral surveillance and public health preparedness.

## 4 MATERIALS AND METHODS

This section provides an overview of the datasets used in this study and presents a detailed description of the proposed TEAM framework, which consists of three key components: (1) a novel time‐enhanced phylogenetic sampling method, (2) feature representation using ESM embeddings, and (3) a DA‐RNN prediction model tailored for multi‐class mutation prediction tasks. Additionally, we demonstrate the application of Bayesian optimization for hyperparameter tuning. Figure 1 illustrates the overall workflow of the proposed methodology.

**FIGURE 1:**
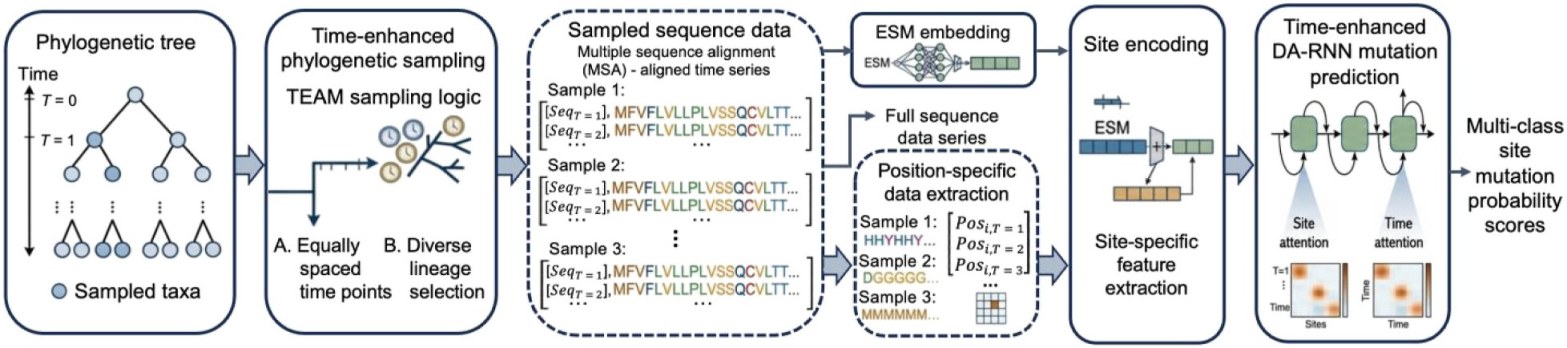
The pipeline of amino acid sequence mutation prediction at specific sites using time‐enhanced attention‐based model (TEAM). The framework integrates time‐enhanced phylogenetic sampling, ESM‐based feature representation, and a dual‐attention recurrent neural network (DA‐RNN) model for multi‐class mutation prediction.

### 4.1 Datasets

#### 4.1.1 Data collection

The SARS‐CoV‐2 spike protein datasets contain a comprehensive collection of approximately 7.62 million SARS‐CoV‐2 sequence entries annotated with PANGO lineages [19]. From this extensive dataset, we focused on sequences sampled from Northern Hemisphere countries during the period from January 2020 to April 2022. Based on this criterion, we sampled about 100,000 sequence IDs that matched the specified time range and geographic region. The corresponding nucleotide sequences were subsequently retrieved from the National Center for Biotechnology Information (NCBI) database and translated into amino acid sequences. This subset provided the foundation for constructing a representative dataset, which was further refined using the time‐enhanced phylogenetic sampling method to generate time‐ordered historical sequences.

#### 4.1.2 Data preprocessing

Multiple sequence alignment was performed using MAFFT [20] (v7.526), aligning each sequence against the standard reference sequence (NC_045512) to ensure a consistent positional reference. Low‐quality sequences and those with incomplete data were filtered out during preprocessing, yielding a high‐quality dataset suitable for downstream analysis. In order to facilitate the subsequent sampling method based on phylogenetic trees, each sequence ID must have time and node information.

### 4.2 Time‐enhanced phylogenetic sampling

The effectiveness of mutation prediction models heavily depends on the quality of the training samples. Existing SARS‐CoV‐2 mutation prediction methods, such as the TEMPO framework, employ PT‐based sampling to extract evolutionary paths. However, these approaches often fail to fully incorporate temporal information, which is particularly important for SARS‐CoV‐2, as its mutation dynamics are influenced by seasonal factors, especially in temperate regions. Moreover, PT‐based sampling methods may introduce a critical issue: the potential for data leakage. Specifically, these methods may inadvertently use information from future time points within an evolutionary path to predict mutations at earlier time points. This can artificially inflate prediction performance by introducing unrealistically high correlations between training and testing samples.

To overcome these limitations, we propose the time‐enhanced phylogenetic sampling method, as outlined in Algorithm 1. This method constructs historical sequences by integrating phylogenetic and temporal constraints to ensure that the training samples capture both evolutionary and temporal dynamics. Specifically, we begin by performing depth‐first search on the PT, as described in Algorithm 2, to extract all evolutionary paths, where each path represents a sequence of nodes from the root to a leaf. For each evolutionary path, multiple samples are then generated by sequentially selecting amino acid sequences that meet both lineage and time interval constraints. If no valid sequence exists for a given time interval, a placeholder (e.g., NA) is inserted to maintain the temporal structure. This ensures that each sample strictly adheres to the chronological order and lineage of the evolutionary path, with the mutation site to be predicted always corresponding to the most recent time point in the sequence. By following this approach, our method eliminates the risk of data leakage and enables the creation of training and testing datasets that are split by time, ensuring robust and meaningful evaluations.

The length of the historical sequences generated by our method differs from those produced by other sampling approaches. For instance, while TEMPO primarily generates sequences of lengths between 4 and 8 due to the shallow PT, our method introduces additional temporal constraints, leading to longer sequences that better reflect mutation dynamics over time. To ensure fair comparisons across different methods, we normalize the sequence lengths by retaining only the last *k* non‐empty sequences from each sample, where *k* is consistent with the settings used in other methods. This adjustment avoids potential biases caused by differences in dataset size while preserving the essential temporal and evolutionary characteristics of the samples.

While this sampling method does not directly capture seasonally driven mutation patterns, it ensures that mutation information is promptly captured at predefined time points. By including time labels in the sampled data, the subsequent prediction model is equipped to learn and identify temporal trends, such as seasonally driven mutation patterns, during the training process. This integration of temporal information enables the framework to provide a more comprehensive and realistic representation of SARS‐CoV‐2’s evolutionary and temporal dynamics.

The selection of the time interval *Δt* and the historical step *k* is informed by the evolutionary characteristics and data availability of the specific viruses. For SARS‐CoV‐2 (spike protein), we utilized a quarterly interval (*Δt* = 3 months). While SARS‐CoV‐2 evolves rapidly, a quarterly resolution provides a robust balance between capturing significant antigenic shifts and maintaining a sufficient sequence density within each interval to avoid excessive data sparsity. For the influenza (HA and NA) datasets, an annual interval was chosen, reflecting the longer seasonal cycles and the traditional surveillance patterns of influenza viruses.

In our experiments, we set *k* = 5, which allows the model to look back at 15 months of evolutionary history for SARS‐CoV‐2 and 5 years for influenza. This window is sufficient to capture the transition between major lineages while adhering to a strict temporal split between training and testing sets to prevent data leakage. When adapting TEAM to other pathogens, *Δt* and *k* can be adjusted: for highly monitored and fast‐evolving viruses, narrower intervals can be employed, whereas for datasets with lower sampling frequency, wider intervals ensure the statistical meaningfulness of the captured mutation patterns.

Algorithm 1 **Time‐enhanced phylogenetic sampling method**

1
Input: Phylogenetic tree *PT*, original dataset *S* (containing separate sequences), number of samples *N* per path, time intervals *Δt*, and dates *D*.
Output: Training and testing dataset D′ (consisting of time‐ordered historical sequences of separate paths).
1:  *D′* ← {}
2:  *Paths* ← DFS(*PT*)
3:  for each *path* ∈ *Paths* do
4:    for *i* = 1 to *N* do
5:      Initialize *d* ← ∅
6:      Extract *path_nodes* from *path*
7:      Sort *path_nodes* by time
8:      for each *node* ∈ *path_nodes* do
9:      Extract *M* from *S* where *M* matches the lineage of node
10:      if *M* is empty then
11:       Continue to next node
12:      else
13:       Randomly select *x* ∈ *M*
14:       Append (*x*, $t_{x}$, lineage) to *d*, where $t_{x}$ is the time of *x*
15:      end if
16:     end for
17:     Generate an empty time series *T* based on *D* and Δ*t*
18:     for each *Δt* ∈ *T* do
19:       if *Δt* is not covered by *d* then
20:        Extract *M* from *S* where *Δt* matches the time and lineage of path
21:        if *M* is empty then
22:        Append (*NA*, *Δt*) to d
23:       else
24:       Randomly select *x* ∈ *M*
25:       Append (*x*, *Δt*, lineage) to *d*
26:       end if
27:     end if
28:    end for
29:    *D*′.add(*d*)
30:   end for
31:  end for
32:  return D′

Algorithm 2 **Depth‐first search (DFS) for path extraction**

1
Input: Phylogenetic tree *PT* (with root node *r*)
Output: Set of paths *Paths* (list of all evolutionary paths from root to leaves)
1:  *Paths* ← ∅ ▷ Initialize an empty set to store paths
2:  procedure DFS(*node*, *current_path*)
3:    Append *node* to *current_path*
4:    if *node* is a leaf then
5:     *Paths* ← *Paths* ∪ copy(*current_path*)
6:    else
7:     for all *child* ∈ children(*node*) do
8:       DFS(*child*, *current_path*)
9:     end for
10:    end if
11:    Remove node from *current_path* ▷ Backtrack to previous node
12:  end procedure
13:  DFS(*r*, ∅)   ▷ Start DFS from the root node with an empty path
14:  return *Paths*

### 4.3 Feature representation with ESM embeddings

To capture the complex structural and functional relationships within protein sequences, we leverage ESM [13, 21] for feature representation. Unlike traditional one‐hot encoding or *k*‐gram methods, ESM is a pre‐trained PLM based on the Transformer architecture. It effectively captures both local and global dependencies by learning high‐dimensional embeddings through masked language modeling on massive protein datasets.

In our framework, we employ the final‐layer hidden states of the ESM model as input features. Each amino acid $x_{i}$ in the sequence is mapped to a contextual embedding $h_{i}^{\left(L\right)}\in R^{d}$, which encapsulates rich evolutionary and structural information. This approach eliminates the need for manual feature engineering and provides a robust representation of the biochemical context, which is critical for the downstream dual‐attention mechanism to capture temporal mutation dynamics.

### 4.4 DA‐RNN prediction model with time embeddings

Our enhanced DA‐RNN integrates time embeddings to explicitly capture temporal dynamics. The unique structure of our dataset and the task requirements, predicting amino acid substitutions within both evolutionary and temporal contexts, necessitated several key modifications to the original DA‐RNN framework. These modifications include the incorporation of time embeddings, input attention specifically designed for high‐dimensional ESM embeddings, and a reconfigured LSTM encoder to model the interplay between evolutionary and temporal dynamics. The framework of the time‐enhanced DA‐RNN is illustrated in Figure 2.

**FIGURE 2:**
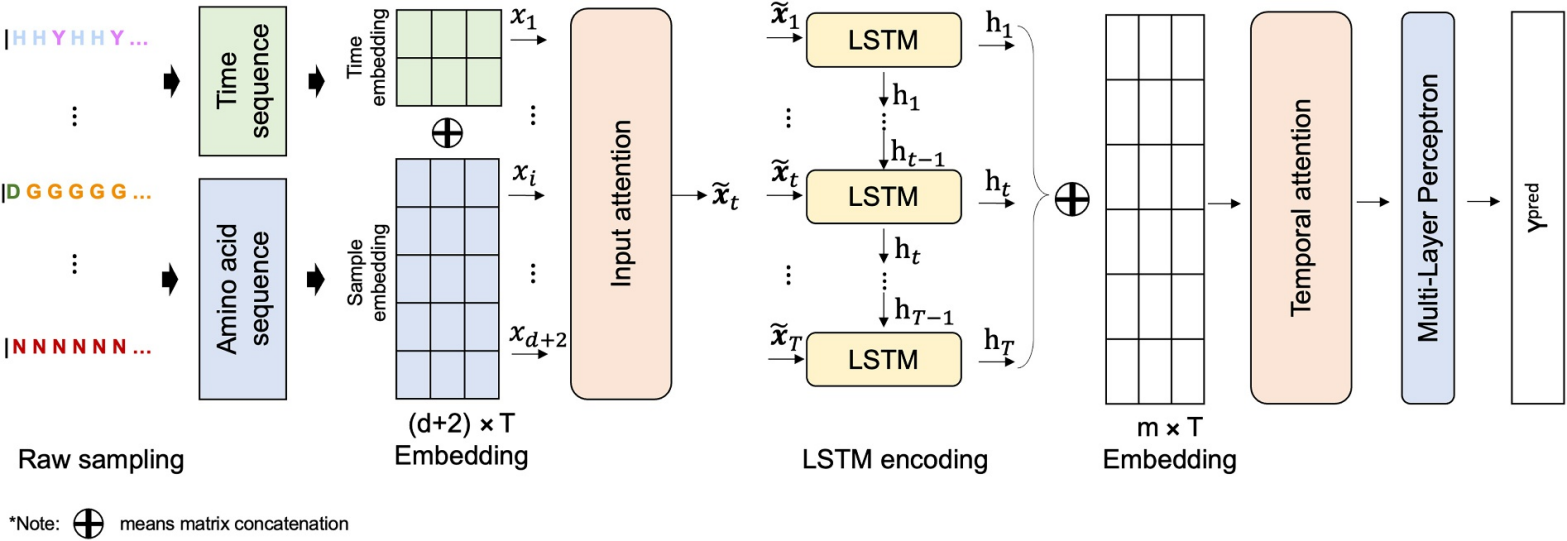
Overview of time enhanced dual‐attention recurrent neural network (DA‐RNN). The model incorporates time embeddings, an input attention mechanism tailored for high‐dimensional evolutionary scale modeling (ESM) embeddings, and a modified long short‐term memory (LSTM) encoder to jointly capture evolutionary and temporal dynamics for amino acid substitution prediction.

To provide a comprehensive understanding of our approach, we now introduce the core components of the time‐enhanced DA‐RNN model. Specifically, we detail the roles of time embeddings, input attention, the LSTM encoder, and temporal attention, and explain how these components work together to align with our goal of predicting mutations at the final time step.

#### 4.4.1 Time embeddings

To integrate temporal information, we append a time embedding $t_{t}$ to the original input $x_{t}$. The time embedding captures periodic trends (e.g., seasonal or quarterly cycles) and is defined as follows:

(3) $$\begin{array}{c} t_{t}=\left[\begin{array}{c} \sin\left(2\pi t/T\right)\\ \cos\left(2\pi t/T\right) \end{array}\right],t_{t}\in R^{2}, \end{array}$$

where *T* is the cycle length (e.g., yearly or quarterly). The input becomes the following:

(4) $$\begin{array}{c} x_{t}^{\text{concat}}=\left[x_{t};t_{t}\right]\in R^{d+2}, \end{array}$$

where $x_{t}\in R^{d}$ represents the amino acid embedding at timestep *t*, derived from ESM. This augmented representation ensures that both sequence features (from ESM embeddings) and temporal trends are incorporated into the model’s input.

#### 4.4.2 Input attention

Given the high dimensionality (1280 dimensions) and potential redundancy of ESM embeddings, the input attention layer assigns weights $\alpha_{t,k}$ to each feature at timestep *t*. The weighted input ${\tilde{x}}_{t}$ is computed as follows:

(5) $$\begin{array}{c} e_{t,k}=v_{e}^{⊤}\;\tanh\left(W_{e}\left[h_{t-1};c_{t-1}\right]+U_{e}x_{t}^{\text{concat},k}\right),\alpha_{t,k}=\text{softmax}\left(e_{t,k}\right), \end{array}$$

here, $e_{t,k}$ denotes the raw attention score for the *k*‐th feature at timestep *t*; $v_{e}$ is a learnable projection vector, $W_{e}$ and $U_{e}$ are trainable weight matrices of the input attention module. This step is crucial for filtering noise in PLM‐derived embeddings and focusing on mutation‐relevant dimensions. This mechanism is particularly crucial for ESM embeddings, as it identifies and prioritizes the most informative features among the 1280 dimensions.

#### 4.4.3 LSTM encoder

To effectively model temporal dependencies in the mutation prediction task, we employ a LSTM based encoder. The LSTM processes the context‐aware representation ${\tilde{x}}_{t}$ (derived from concatenated ESM and time embeddings) across *T* timesteps. At each timestep *t*, the encoder updates its hidden state $h_{t}$ and cell state $c_{t}$ to capture both short‐term evolutionary context and long‐term temporal trajectories as follows:

(6) $$\begin{array}{c} h_{t},c_{t}=\text{LSTM}\left({\tilde{x}}_{t},h_{t-1},c_{t-1}\right), \end{array}$$

where $h_{t},c_{t}\in R^{m}$. The sequence of hidden states across all timesteps forms the encoded output $H=\left[h_{1},h_{2},\text{…},h_{T}\right]\in R^{T\times m}$.

By maintaining information from previous time intervals, the LSTM encoder enables the model to effectively link sequence‐level patterns with the cumulative evolutionary dynamics of SARS‐CoV‐2. This design ensures that the final prediction at the target timestep is informed by the entire historical trajectory of amino acid substitutions.

#### 4.4.4 Temporal attention

To identify the most influential time points in the evolutionary trajectory, a temporal attention mechanism computes weights over the encoder’s hidden states H. The relevance score $l_{t}^{i}$ for each timestep i is determined by the alignment between the encoder state $h_{i}$ and the previous decoder state $d_{t-1}$

(7) $$\begin{array}{c} l_{t}^{i}=v_{d}^{⊤}\;\tanh\left(W_{d}h_{i}+U_{d}d_{t-1}\right),\beta_{t}^{i}=\text{softmax}\left(l_{t}^{i}\right). \end{array}$$

The resulting context vector ${\text{cv}}_{t}=\sum_{i=1}^{T}\beta_{t}^{i}h_{i}$ provides a weighted summary of the sequence, effectively allowing the model to focus on critical evolutionary transition points. This mechanism ensures that the decoder prioritizes timesteps that are most predictive of the final mutation outcome.

#### 4.4.5 Prediction layer

The context vector ${\text{cv}}_{t}$, which encapsulates the weighted temporal and evolutionary information, is combined with the decoder state to generate the final prediction. At each decoding step, the decoder state $d_{t}$ is updated via another LSTM cell as follows:

(8) $$\begin{array}{c} d_{t}=\text{LSTM}\left(\left[y_{t-1};{\text{cv}}_{t}\right],d_{t-1}\right), \end{array}$$

where $y_{t-1}$ is the prediction from the previous timestep. Finally, a linear transformation is applied to the decoder state $d_{t}$ to compute the probability distribution of amino acid substitutions as follows:

(9) $$\begin{array}{c} {\hat{y}}_{t}=W_{y}d_{t}+b_{y}, \end{array}$$

where $W_{y}$ denotes the learnable weight matrix of the output linear layer, and $b_{y}$ is the corresponding bias term.

Our enhanced dual attention model is tailored to our mutation prediction task, where each sample consists of the ESM embeddings of a specific amino acid across multiple time intervals. The integration of time embeddings, input attention for high‐dimensional ESM embeddings, and the dual‐state mechanism of the LSTM allows the model to effectively learn temporal and evolutionary dynamics. This ensures accurate predictions of amino acid substitutions at the final time point in each sample, addressing the challenges posed by SARS‐CoV‐2’s rapid evolution.

### 4.5 Bayesian optimization for hyperparameter tuning

To ensure a fair comparison and optimal performance for all models, including DA‐RNN and baseline models (RNN, LSTM, and GRU), we employed Bayesian optimization for hyperparameter tuning. Bayesian optimization is a sequential optimization method that constructs a probabilistic model to approximate the objective function and uses it to determine the next point to evaluate. This approach is particularly suitable for optimizing expensive‐to‐evaluate functions, such as training deep learning models.

Let f(x) denote the objective function, where x represents the hyperparameter configuration (e.g., learning rate, hidden layer size). Our goal is to find the hyperparameter set $x^{*}$ that maximizes the validation performance, where X is the search space of hyperparameters.

(10) $$\begin{array}{c} x^{*}=\arg\max_{x\in X}f\left(x\right). \end{array}$$

In Bayesian optimization, a Gaussian process (GP) is used as the surrogate model to approximate f(x). A *GP* defines a distribution over functions and is parameterized by a mean function μ(x) and a kernel function $k\left(x,x^{\prime }\right)$.

(11) $$\begin{array}{c} f\left(x\right)∼GP\left(\mu \left(x\right),k\left(x,x^{\prime }\right)\right). \end{array}$$

The acquisition function $\alpha_{\text{EI}}\left(x\right)$ determines the next point to evaluate by balancing exploration (sampling in less certain regions) and exploitation (sampling near the current optimum). Common acquisition functions include the following:

(12) $$\begin{array}{c} \alpha_{\text{EI}}\left(x\right)=E\left[\max\left(f\left(x\right)-f\left(x^{+}\right),0\right))\right], \end{array}$$

where $f\left(x^{+}\right)$ is the current maximum observed value, and E denotes the expectation.

The Bayesian optimization process iteratively updates the surrogate model and acquisition function based on the observed data:

1. Initialize by randomly sampling a few hyperparameter configurations and evaluating their performance.
2. Fit the *GP* model to the observed data.
3. Optimize the acquisition function to propose the next hyperparameter configuration.
4. Evaluate the performance of the proposed configuration and update the observed data.
5. Repeat steps 2–4 until the stopping criterion is met (e.g., maximum iterations or convergence achieved).

For each model, the search space included the following:

- Learning rate: [10^−5^, 10^−1^] (logarithmic scale)
- Hidden layer size: {64, 128, 256, 512}
- Dropout rate: [0.1, 0.5]
- Batch size: {16, 32, 64, 128}

We used the *scikit‐optimize* library to implement Bayesian optimization and evaluated each configuration using 5‐fold cross‐validation on the training set. The final hyperparameter configuration was selected based on the highest average validation accuracy.

This approach ensures a fair comparison by evaluating all models under their respective optimal configurations, thereby mitigating the impact of suboptimal hyperparameter selection. To validate the reliability of this optimization, we performed 10 independent trials across all datasets. The results of optimal hyperparameters were highly concentrated, with negligible performance variance (e.g., for TEAM on the SARS‐CoV‐2 S protein dataset, MCC = 0.8338 ± 0.0020), underscoring the reproducibility and robustness of our tuning pipeline.

## AUTHOR CONTRIBUTIONS

**Jie Ji:** Conceptualization; methodology; software; data curation; formal analysis; visualization; writing—original draft; writing—review and editing. **Jie Hu:** Writing—review and editing. **Tianwei Yu:** Resources; supervision. **Xiaodan Fan:** Supervision; funding acquisition; project administration.

## CONFLICT OF INTEREST STATEMENT

The authors declare no conflicts of interest.

## ETHICS STATEMENT

All sequence datasets utilized in this manuscript were retrieved from publicly accessible repositories, where original data collection had already obtained relevant ethical approval and patient informed consent by the original data submitters. This study only conducts secondary computational analysis on de‐identified, anonymized public data, and no additional human or animal specimen collection was performed by our research team.

## Data Availability

The code and data are publicly available at GitHub website (ddduooo99/TEAM‐covid19).
